# Nutritional composition, lipid profile and stability, antioxidant activities and sensory evaluation of pasta enriched by linseed flour and linseed oil

**DOI:** 10.1186/s12896-024-00841-w

**Published:** 2024-05-15

**Authors:** Zahra Amri, Amira Mnari Bhouri, Madiha Dhibi, Mohamed Hammami, Sonia Hammami, Beligh Mechri

**Affiliations:** Biochemistry Laboratory, Faculty of Medicine, LR12ES05 “Nutrition- Functional Foods and Vascular Health”, University of Monastir, 5019 Monastir, Tunisia

**Keywords:** Pasta, Fortification, Linseeds, Linseeds oil, Antioxidant activity, Sensory evaluation

## Abstract

Pasta assortments fortified with high quality foods are a modern nutritional trends. This study, explored the effects of fortification with linseed flour (LF) and linseed oil (LO) on durum wheat pasta characteristics. Wheat flour semolina was replaced with 5%, 10% and 15% of LF or 1%, 2.5% and 5% of LO. Control pasta CP (without LF or LO addition), LF-enriched pasta LFP 5%, LFP 10% and LFP 15% and LO-enriched pasta LOP 1%, LOP 2.5% and LOP 5% was compared for the proteins, fat and phenolic contents and fatty acids (FA) profile. Impact on lipid oxidation and sensory evaluation were also determined*.* Fortification of pasta with LF improved significantly (*p* < 0.05) the contents of protein, fat and phenolic compared to CP whereas the enrichment of pasta with LO resulted in a significant increase (*p* < 0.05) in the content of fat and a significant decrease in protein and phenolic contents. All the formulations decreased the saturated FA percent and increased the polyunsaturated FA percent with enhancement of omega-3 FA content. Antioxidant activity measured by FRAP and DPPH assays was improved after the fortification. For lipid oxidation, the replacement of semolina by LF or LO promoted an increase (*p* < 0.05) on TBARS values in level-dependent manner. Regarding sensory evaluation, the two types of fortification did not affect the taste; flavor and aroma of cooked pasta, but LOP 5% showed the highest score of the overall acceptability. The results recommended the possibility of producing pasta supplemented with LF or LO (even at a level of 15% and 5% respectively) as a functional food.

## Introduction

Natural bioactive compounds and extracts have long been known to have health benefits [1, 2] In fact, most of the bioactive compounds have antioxidant, anticarcinogenic, antiinflammatory, [3] and antimicrobial effects [4]. Today, These compounds naturally found in various foods, are gaining interest in food industry due to changes in consumer habits and they may be added as natural additives in foods [5–7]. Dietary linseeds or flaxseed is one of these foods that contain several healthful components predominantly oil (40–45%), especially ω-3 fatty acid (α-linolenic acid (ALA)), lignan (1%) especially secoisolariciresinol diglycoside (SDG) and dietary fiber (20–25%) mucilage [8]. It contains also other less abundant components including the proteins like cysteine, methionine and arginine, phenolic acids, flavonoids and potassium which have health benefits. ALA is an omega-3 fatty acid and has been reported to be useful in the prevention of the cardiovascular disease by the reduction of two risk factors hypertension and high cholesterol levels [9]. SDG is the most prevalent lignan in linseeds and are characterized as phenolic acid and it act as both antioxidants and phytoestrogens [10]. Linseeds fiber has hypocholesterolemic effects and reduce the serum levels of Triglycerides [11]. Due to the high nutritional content and health benefits, linseeds ingested in clinical trials may be administered in capsule form [12], or fortified in functional foods like yogurt [5] or supplemented other cereal based products, including bread [13, 14], pasta [6] and noodles [15]. However, the physicochemical properties of dried pasta manufactured with partial replacement of semolina by linseed flour and/or linseed oil is still little known. In this context, and to better explore the nutritional role of linseeds in pasta production, we investigate in the present study the optimization levels of linseeds flour (LF) and linseeds oil (LO) to improve the nutritional and sensory values of the product. Firstly, LF and LO were compared for their phenolic content and antioxidant activities. Then, for pasta production, durum wheat semolina pasta with 3 substitution levels of LF, being 5, 10, and 15 g/100 g (w/w) or 3 substitution levels of LO, being 1, 2.5, and 5 g/100 g (w/w). After pasta fabrication, we evaluated the effect of increasing levels of LF or LO in durum wheat semolina pasta by focusing on (i) nutritional and fatty acids composition, (ii) Antioxidant and anti-lipid oxidation impacts and (iii) the sensory evaluation of different cooked pasta samples.

## Material and methods

### Materials

Linseeds and linseeds oil (LO) extracted by supercritical CO2 were purchased from society of.

NATURLINA PRECIEUX (Bullarigeéa, 8100 Jendouba, Tunisia). Pure durum wheat Semolina was obtained from Society of pasta *Warda*, Sousse, Tunisia. All chemicals reagents were of analytical grade, and were acquired from Sigma Aldrich.

### Linseeds flour (LF) preparation

LF were sundried until constant weight. Then the sundried seeds were finely ground using a Moulinex grinder (Moulinex LM242027, 450 watts, France) at high speed for 3 min with particle size of 0.5 mm and stored immediately in polypropylene package at 4 °C until used.

### Pasta making

Pasta flour was prepared by mixing durum wheat semolina (SE) and LF at level of 5%, 10% and 15% (w/w) of SE or by mixing SE and LO at levels of 1%, 2.5% and 5% of SE. For each formulation, pasta flour was manually mixed with tap water (flour: water, 1:0.4, w/w) 5 min to obtain a homogeneous dough. Pasta samples were referred to as CP, control pasta without supplementation, LFP 5%, LFP 10% and LFP 15%, respectively pasta enriched with 5%, 10% and 15% of LF and LOP 1%, LOP 2.5% and LOP 5%, respectively pasta enriched with 5%, 10% and 15% of LO. The dough was fabricated using laboratory scale extruder (TR 70 INOX version, Lineapasta equipment) equipped with bronze dies (Diameter 70 mm) and the vat capacity is 2.5 kg. Fresh pasta (ziti shape) was dried at 50 °C for 14 h in a hot air drier. Finally, the pasta samples were covered by the polypropylene package and were kept at room temperature for the next analysis.

### Phytochemicals screening

Total Phenolic Content (TPC) of raw materials (LF and LO) and different pasta samples was quantified according to the method of Montedoro et al. [16] and was expressed as mg of gallic acid equivalent per g of dried weight (mg GAE/g DW). Fat (method 30–10.01) and protein (method 46–10.01) contents of pasta samples were determined in triplicates according to American Association of Cereal Chemists, 2000 [17].

### Fatty acids profile

The dried pasta samples were ground and then oils was extracted with hexane using a Soxhlet system. For total fatty acid analysis, extracted oils were converted to fatty acid methyl esters (FAMEs) and injected on a Hewlett-Packard gas chromatograph (Hewlett-Packard, Palo Alto, CA) according the procedure described by Mekni et al. [18]. Then, FAMEs were identified by comparing their relative and absolute retention times to those of authentic fatty acid standards analyzed under the same conditions and were quantified according to their percentage area in the lipid fraction.

### Antioxidant activity

The DPPH method was used to determine antioxidant activity of samples using 1,1-diphenyl-2-picrylhydrazyl (DPPH) free radical as a reagent according to Braca et al. [19]. The ferric reducing antioxidant potential (FRAP) was determined according to the method described by Oyaizu et al. [20]. In each assay, the absorbance was measured by LAMBDA 365 UV–Vis Spectrophotometer and results were expressed in terms of inhibition percent.

### Lipid oxidation

Lipid oxidation was determined using the protocol of the thiobarbituric acid-reactive substances (TBARS) proposed by Yin et al. [21]. Five grams of each sample was mixed with 22.5 mL of 11% trichloroacetic acid for 1 min in an Ultra-Turrax (IKA T25 digital) followed by 1 min in an ice bath, and another 1 min of homogenization at equal conditions. The homogenate was filtered using Whatman paper N˚1, and 1 mL of 20 mM TBA was added to 1 mL of the filtrate following incubation in dark conditions for 20 h at 25˚C. TBARS analyze was performed in triplicate. The absorbance value at 532 nm was read using a UV-1800 spectrophotometer (Shimadzu, Kyoto, Japan) and the results were expressed as TBARS number.

### Sensory assessment

Pasta samples were cooked in distilled water (pasta: water ratio is 1:4 gmL^−1^) to optimum cooking time (between 7–8 min) and served to the panelists. The sensory test panel consisted of eight panelists (4 females and 4 males aged 25–55 years) who were trained academic staff. Panelists judged flavor, taste, odor and overall acceptability using a 10-point hedonic scale ranging from 10–5 (like extremely) to 4–1 (dislike extremely) for each sensory characteristic. The score for each sensorial component was the average of three individuals’ assessments [22].

### Statistical analysis

The results were expressed as mean ± standard deviation of three independent experiments (*n* = 3). Analyses of variance were performed by the one-way ANOVA. Procedure and significance of differences was verified by Tukey's multiple range test (*P* < 0.05). ANOVA analyses were performed using the statistical package SPSS version 21 (Statistical Package for Social Science, SPSS Inc., Chicago, IL).

## Results and discussion

### Total phenolic content (TPC) and antioxidant activity of linseeds flour and oil

Table 1 showed TPC of linseeds flour LF and oil LO and the IC_50_ values which correspond to the amount of extract required to scavenge 50% of DPPH radicals present in the reaction mixture. LF showed higher TPC at 279.5 ± 30 mg GAE/g DW and an IC_50_ value of 2.28 ± 0.04 mg/ml. The lipid faction of linseeds LO contained also a phenolic content estimated by 20 ± 0.04 mg GAE/g DW and exhibited an antioxidant activity with IC_50_ value of 0.16 ± 0.01 mg/ml. Our results concerning LO are similar to that found by Nazia et al. [23] who reported that TPC of linseeds cultivars, native to Pakistan ranged from 2560 to 3286 mg gallic acid equivalent (GAE)/100 g. It has been reported that lignans are the major phenols present in linseed and also in linseeds oil but in relatively low levels [3]. These molecules are known as power antioxidants and display as sequestrators of hydroxyl radicals [24].

**Table 1:** Phenolic content and antioxidant activity of linseeds flour (LF) and Oil (LO)

	LF	LO
TPC (mg GAE/ g DW)	279.5 ± 30^a^	20 ± 2^b^
DPPH activity IC_50_ (mg/ml)	2.28 ± 0.04^a^	0.16 ± 0.01^b^

Each value represents the mean of three determinations (*n* = 3) ± standard deviation

^a,b^Means followed by the letters within the same row indicate significant difference (*p* < 0.05) in Tukey test

### Chemical composition of pasta samples

Table 2 reports the lipids, protein and TPC of control and supplemented pasta. LF incorporation increased slightly the protein content and highly the lipids compared to CP whereas LO addition decreased the protein content and increased highly the lipid content potentially due to the LO composition. In fact, it has been reported that Whole and ground linseed is rich in fat (42%), protein (18%) and fiber (27%) with minimal vitamin and mineral however, flax oil in its natural state is 100% fat, 53% of which is ALA, has low protein content and is devoid of carbohydrates and dietary fiber [12, 25]. Linseed proteins contain rich proportions of amino acids such as glutamic acid, methionine, arginine, cysteine and aspartic acid, with low amounts of lysine, threonine and tyrosine [26] and they have been associated with antifungal properties and antioxidant activities.

**Table 2:** Composition of control pasta (CP) and pasta samples enriched with linseeds flour (LFP) and linseeds oil (LOP)

	**CP**	**LFP**			**LOP**		
		LFP 5%	LFP 10%	LFP 15%	LOP 1%	LOP 2.5%	LOP 5%
Protein (%)	12.07 ± 0.12^c^	12.37 ± 0.02^b^	12.62 ± 0.02^b^	12.89 ± 0.02^a^	11.87 ± 0.04^ cd^	11.61 ± 0.01^d^	11.33 ± 0.15^e^
Lipids (%)	0.66 ± 0.05^d^	1.67 ± 0.15^c^	2.85 ± 0.13^b^	5.74 ± 0.25^a^	1.69 ± 0.31^c^	4.40 ± 0.17^b^	6.41 ± 0.37^a^
TPC (mg GAE/g DW)	45.10 ± 4.55^bc^	46.21 ± 0.01^bc^	48.4 ± 6.40^b^	60.4 ± 4.20^a^	33.28 ± 0.74^d^	35.47 ± 0.82^d^	38.19 ± 0.82^ cd^

Values are presented as means ± DS. CP, control pasta; LFP 5%, LFP 10% and LFP 15%: pasta prepared with 5, 10 and 15 g of linseeds flour, LOP 1%, LOP 2.5% and LOP 5%: pasta prepared with 1, 2.5 and 5 g of linseeds oil

^a,b,c,d,e^Means followed by the letters within the same row indicate significant difference (*p* < 0.05) in Tukey test

The addition of LF and LO increased lipids contents in pasta by 8–ninefold compared to CP. Lipids are one of the high value functional ingredients in linseeds and are partly the reason why linseeds are considered to be a major health promoting agent. Food Products containing linseed and its derivatives have been proposed as the principal source of omega 3 fatty acids and as nourishing supplements for a range of dietary entities [5, 6, 15]. Manthey et al. 2002, reported that lipid, ALA, and FFA contents declined during the extrusion process in dried spaghetti samples containing semolina fortified with 5 and 10% ground linseed. Moreover, they supposed that this decline in lipid content probably occurred during dough development in the extrusion process [6].

The fortification of pasta with LF improved the phenolic content (TPC) compared to CP. The highest value of 60.4 ± 4.2 mg GAE/g DW was recorded for the LFP 15%. However, pasta fortified with LO showed lower TPC compared to CP. Linseeds polyphenols such as SDG have multiple biological functions such as antioxidant, anti-inflammatory, anti-cancer and antimicrobial activities. Nowadays there is interest in using whole linseed (flour) as functional food ingredients owing to the healthier and more sustainable properties than those of isolated linseed ingredients [27]. Moreover, recent studies suggested that Linseed lignans found in grain flour can be used as a good antioxidant for improving the stability of linseed oil incorporated into functional foods [28].

### Fatty acids profiles of pasta samples

**Fig. 1:**
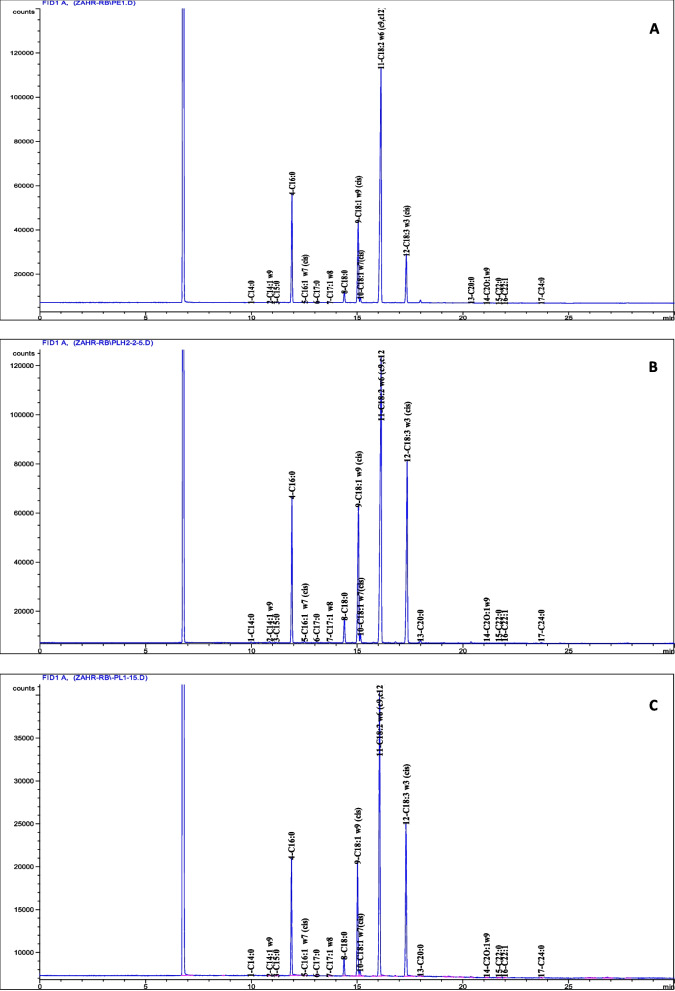
Typical chromatogram of fatty acids analyzed by GC in control pasta (**A**), linseeds oil enriched-pasta LOP 2.5% (**B**) and linseeds flour enriched-pasta LFP 15% (**C**)

The fatty acid composition of the pasta samples is presented in Table 3 and Fig. 1. A total of seventeen fatty acids were identified in fortified pasta samples. Generally, all the formulations showed significance difference (*p* < 0.05) in total SFA and PUFA compared to the control pasta. LF and LO fortification increased significantly the PUFA percent particularly the omega-3 fatty acids (C18:3) amount and decreased the SFA percent.

**Table 3:** Fatty acids composition (%) of different pasta samples

	**CP**	**LFP 5%**	**LFP 10%**	**LFP 15%**	**LOP 1%**	**LOP 2.5%**	**LOP 5%**
*C14:0*	0.09 ± 0.00	0.05 ± 0.02	0.05 ± 0.02	0.07 ± 0.02	0.09 ± 0.00	0.07 ± 0.01	0.06 ± 0.02
*C14:1 w9*	0.12 ± 0.00	0.10 ± 0.03	0.1 ± 0.03	0.08 ± 0.03	0.08 ± 0.00	0.07 ± 0.01	0.07 ± 0.01
*C15:0*	0.04 ± 0.00	0.04 ± 0.01	0.04 ± 0.01	0.03 ± 0.01	0.04 ± 0.01	0.03 ± 0.01	0.03 ± 0.02
*4-C16:0*	20.60 ± 0.02	13.54 ± 0.68	13.12 ± 0.67	12.06 ± 0.68	13.32 ± 1.14	13.06 ± 0.46	12.05 ± 1.01
*C16:1 w7 (cis)*	0.17 ± 0.00	0.19 ± 0.08	0.19 ± 0.08	0.13 ± 0.08	0.12 ± 0.01	0.13 ± 0.02	0.12 ± 0.01
*C17:0*	0.10 ± 0.00	0.12 ± 0.04	0.12 ± 0.03	0.09 ± 0.04	0.08 ± 0.00	0.08 ± 0.00	0.08 ± 0.01
*C17:1 w8*	0.12 ± 0.00	0.08 ± 0.01	0.1 ± 0.013	0.08 ± 0.01	0.07 ± 0.00	0.06 ± 0.01	0.07 ± 0.01
*C18:0*	0.94 ± 0.01	2.48 ± 0.05	2.41 ± 0.05	2.52 ± 0.05	2.61 ± 0.33	2.59 ± 0.35	2.53 ± 0.41
*C18:1 w9 (cis)*	18.05 ± 0.05	15.73 ± 0.18	15.23 ± 0.17	14.85 ± 0.18	15.75 ± 0.21	15.50 ± 0.45	14.44 ± 0.39
*C18:1w7(cis)*	1.06 ± 0.00	0.88 ± 0.04	0.85 ± 0.04	0.85 ± 0.04	0.9 ± 0.09	0.92 ± 0.09	0.79 ± 0.1
***C18:2 w6 (c9,c12)***	53.60 ± 0.16^a^	41.67 ± 0.31^b^	40.37 ± 0.29^c^	39.36 ± 0.31^d^	41.64 ± 0.31^b^	40.26 ± 0.31^c^	38.53 ± 0.51^d^
***C18:3 w3 (cis)***	4.42 ± 0.16^d^	24.54 ± 0.24^c^	26.90 ± 0.25^b^	28.84 ± 0.16^a^	25.02 ± 0.84^c^	27.33 ± 1.16^b^	29.58 ± 0.51^a^
*C20:0*	0.23 ± 0.01	0.32 ± 0.00	0.31 ± 0.00	0.32 ± 0.00	0.31 ± 0.01	0.34 ± 0.05	0.22 ± 0.14
*C2O:1w9*	0.10 ± 0.01	0.05 ± 0.00	0.1 ± 0.003	0.05 ± 0.00	0.05 ± 0.01	0.05 ± 0.01	0.04 ± 0.01
*C22:0*	0.05 ± 0.01	0.03 ± 0.01	0.03 ± 0.01	0.04 ± 0.01	0.04 ± 0.05	0.05 ± 0.01	0.05 ± 0.01
*C22:1*	0.10 ± 0.01	0.06 ± 0.04	0.06 ± 0.04	0.09 ± 0.04	0.02 ± 0.00	0.06 ± 0.03	0.06 ± 0.03
*C24:0*	0.19 ± 0.01	0.11 ± 0.02	0.11 ± 0.02	0.12 ± 0.02	0.08 ± 0.01	0.07 ± 0.06	0.1 ± 0.02
***∑SFA***	22.29 ± 0.06^a^	16.70 ± 0.45^bc^	16.18 ± 0.36^bc^	15.72 ± 0.41^bc^	16.6 ± 0.82^bc^	16.29 ± 0.51^bc^	15.12 ± 0.73^c^
***∑PUFA***	58.02 ± 0.00^c^	66.21 ± 0.39^b^	67.27 ± 0.31^ab^	68.20 ± 0.33^a^	66.66 ± 1.16^ab^	67.25 ± 0.39^ab^	68.11 ± 1.01^a^

Values are presented as means ± DS. CP, control pasta; LFP 5%, LFP 10% and LFP 15%: pasta prepared with 5, 10 and 15 g of linseeds flour, LOP 1%, LOP 2.5% and LOP 5%: pasta prepared with 1, 2.5 and 5 g of linseeds oil.

^a,b,c,d^Means followed by letters within the same row indicate significant difference (*p* < 0.05) in Tukey test

Regarding SFA, the decrease is estimated by 30% in pasta manufactured with LF 15% and LO 5% compared to CP and the palmitic acid (C16:0) was the major SFA. For MUFA, a significant decrease in oleic acid C18:1 w9 was recorded from 18 ± 0.05% in CP to 14 ± 0.18% in LFP15% and 14.44 ± 0.39% in LOP 5%. The PUFA fraction presented 66–68% of the composition of the pasta enriched by LF and LO. A remarkable decrease in linoleic acid C18:2 w6 was also showed in enriched pasta (from 53.60 ± 0.16% in CP to 39.36 ± 0.31in LFP15% and 38.53 ± 0.51% in LOP5% fortified pasta samples at highest dose. However, the percent of omega-3 fatty acid, the linolenic acid was enhanced compared to that of CP and it increased by sevenfold. These findings are consistent to that found by Manthey et al., 2002 who showed that ALA content in spaghetti made from semolina fortified with ground linseed increased with increased ground linseed concentration. Whereas linoleic acid was the predominant fatty acid in lipid extracted from semolina (54.9%) followed by palmitic acid (20%) [6]. Same findings were showed in bakery and dairy foods. In fact, the partial substitution of soybean oil with linseed oil in the preparation of whole-wheat linseed bread enriched with omega-3 increased the omega-3 percentage in formulations, which improved the nutritional value of whole-wheat linseed rolls enriched with LO [13]. the addition of linseed powder in yogurt formulation significantly decreased the saturated fatty acids (SFAs), the ratio of omega-6 to omega-3 fatty acids, and atherogenic indices of yogurts and increased the polyunsaturated fatty acids (PUFAs) [5].

### Antioxidant activities

**Fig. 2:**
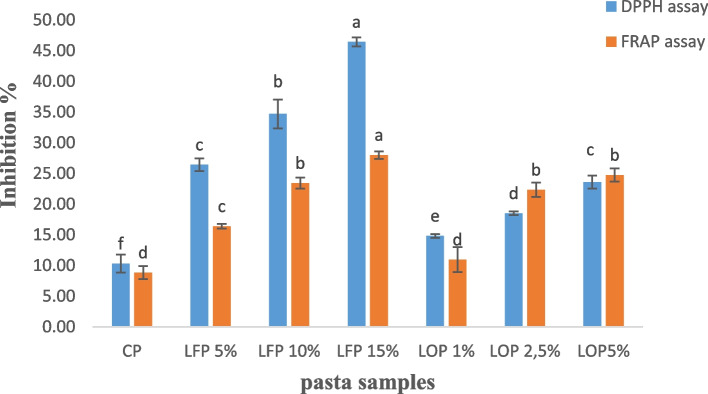
DPPH and FRAP assays (inhibition %) in control pasta (CP), pasta fortified with different linseeds flour levels (LFP 5%, LFP 10% and LFP 15%) and linseeds oil (LOP 1%, LOP 2.5% and LOP 5%). Results are expressed as means ± standard deviation (*n* = 3). Different letters indicate significance differences (*p* < 0.05) among pasta samples according to Tukey’s test

The percentage inhibition of DPPH and FRAP measured the free radical scavenging attributes of the different pasta samples and indicate its antioxidant potential. The antioxidant inhibition of pasta ranged from 8 to 50% (Fig. 2). Significant improvement in both the parameters was observed in comparison to control pasta, and maximum inhibition was recorded for LFP 15% followed by LFP 10%. Similarly, the fortification of pasta with LO improved the antioxidant activities in dose dependent manner. These results are in accordance with a previous study carried out on noodles fortified with ground linseed which showed that the in vitro antioxidant activities, measured by FRAP, DPPH and ABTS assays were increased [29]. In addition, Chinese steamed bread containing barley, linseed, and barley–linseed hull extracts showed that antioxidant activity and total phenolic content was increased compared to control [30]. The antioxidant activity of enriched pasta may be attributed to phenolic compounds found in linseeds such as lignan, a hydroxyl radical scavenger. These antioxidant capacity might contribute to a several health benefits, including protective effects against cardiovascular diseases, diabetes, cancer, and mental stress [31].

### Impact on lipid oxidation

**Fig. 3:**
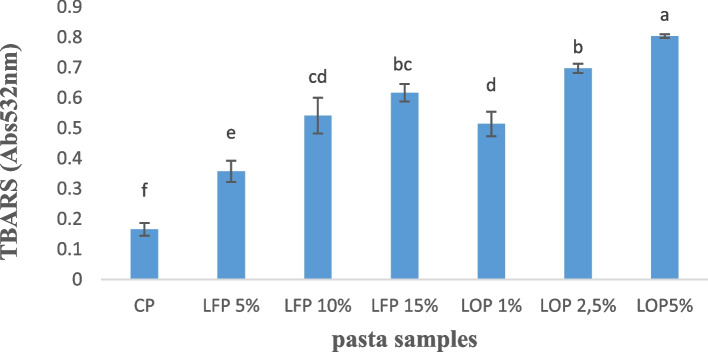
Thiobarbituric acid-reactive substances (TBARS) in control (CP), pasta fortified with different linseeds flour levels (LFP 5%, LFP 10% and LFP 15%) and linseeds oil (LOP 1%, LOP 2.5% and LOP 5%). Results are expressed as means ± standard deviation (*n* = 3). Different letters indicate significance differences (*p* < 0.05) among pasta samples according to Tukey’s test

The replacement of semolina by LF or LO promoted an increase (*p* < 0.05) on TBARS values in level-dependent manner (Fig. 3). Linseeds contain high amount of polyunsaturated fatty acids (PUFA) which are more susceptible to lipid oxidation supporting our findings for the TBARS values. Waszkowiak et al., 2019 suggested that lipid oxidation in linseeds-enriched foods can be attributed to that linseed roasting caused the destruction of the membrane integrity of oil body, thus increasing the direct contact between bulk oil and oxygen [32]. In these cases, the addition of some natural or synthetic antioxidants such as ascorbic acid and butylated can be an alternative solution to reduce oxidation of in linseed-enriched foods.

Lipid oxidation in LOP is higher than that in LFP. This finding can be explicated by that the lipids in linseed are packed into oil bodies, which are protected by their phospholipid-protein membrane. In addition, the intrinsic antioxidants such as phenolic compounds in linseed may hinder the occurrence of lipid oxidation for linseed-enriched products [33]. Recent studies suggested that both Linseed lignan extract and SDG were good plant-based antioxidants for improving the stability of linseed oil nanoemulsions and their incorporation into functional foods and beverages [28]. Our results were not consistent with those reported by Manthey et al. 2002, who showed that the levels of FFA and conjugated dienes, the two parameters of lipid oxidation, were reduced during processing and cooking of spaghetti fortified with ground linseed indicating that the triacylglycerols and ALA remained stable [6]. Moreover, lipid remained relatively stable during processing and cooking of macaroni fortified with 15% of ground linseed [34].

### Sensory evaluation

Generally, enrichment of a food product with bioactive components should not affect its sensory quality. In addition to its health benefits, the product should be very good in terms of taste, aroma and appearance. Results in Table 4 showed that linseed flour or oil could be successfully incorporated into pasta without adversely affecting their sensory attributes. Regarding overall acceptability, LO-enriched pasta with 5% of LO showed the highest score. However, there is no statistical difference between CP, LF-enriched pasta at different levels and LO-enriched pasta at 1% and 2.5%. Wheat bread and biscuits could be enriched by linseed fibre for 5%, but a larger addition could cause adverse sensory evaluation. In other studies, the sensory profiles of bread and other baking products with yellow and brown linseed fibre [35, 36] in the amounts of 2.5% and 5.0% were also acceptable. In another study, linseed at 30–50% substitution for flour greatly enhanced the nutritional qualities of some nutrients such as Linolenic acid, fiber and folate contents without affecting the overall acceptability of bakery products like breads and muffins [37].

**Table 4:** Results of cooked pasta sensory evaluation

	**CP**	**LFP 5%**	**LFP 10%**	**LFP 15%**	**LOP 1%**	**LOP 2.5%**	**LOP 5%**
Flavor	6.28 ± 0.75	6.14 ± 0.69	6.71 ± 0.75	6.28 ± 1.38	7.14 ± 1.21	6.78 ± 1.07	7.33 ± 0.81
Taste	5.85 ± 0.69	6.00 ± 0.81	6.14 ± 0.89	6.42 ± 1.51	7.00 ± 1.15	7.00 ± 1.00	7.57 ± 1.27
Aroma	6.00 ± 0.57	6.71 ± 0.75	6.71 ± 1.80	6.85 ± 1.67	7.00 ± 1.15	6.14 ± 1.07	7.14 ± 1.34
Overall judgment	5.85 ± 0.70^b^	6.42 ± 0.53^ab^	6.14 ± 1.34^ab^	6.85 ± 1.21^ab^	7.66 ± 0.81^ab^	7.57 ± 0.78^ab^	7.28 ± 1.50^a^

Values are presented as means ± DS. CP, control pasta; LFP 5%, LFP 10% and LFP 15%: pasta prepared with 5, 10 and 15 g of linseeds flour, LOP 1%, LOP 2.5% and LOP 5%: pasta prepared with 1, 2.5 and 5 g of linseeds oil

^a,b^Means followed by the letters within the same row indicate significant difference (*p* < 0.05) in Tukey test

## Conclusion

Obtained results showed that linseed flour and oil could be useful additives for durum wheat pasta production with enhanced pro-health properties. En effect, enrichment of durum wheat pasta with the addition of linseed flour and oil resulted in high increase in fat content compared with control pasta with an important enhancement of the percent of omegra-3 fatty acid, the linolenic acid. In addition, enrichment of pasta with LF improved the protein and total phenolic contents and contributed in increasing the antioxidant activity of supplemented pasta. The replacement of wheat flour by LF and LO increased lipid oxidation without negatively affecting the sensory evaluation of pasta. Regarding overall acceptability, LO-enriched pasta with 5% of LO showed the highest score. The addition of natural antioxidant ingredients can protect the stability of enriched products and that warrants further investigation.

## Data Availability

No datasets were generated or analysed during the current study.

## Abbreviations

CaCl_2_: Calcium dichloride
DMSO: Dimethyl Sulfoxide
DPPH: 2,2-Diphenyl-1-picrylhydrazyl
HCl: Chlorhydric acid
TPC: Total Phenol Content
TFC: Total Flavonoids Content
TTC: Total Tannins Content

## References

[1] Banwo K, Olojede AO, Adesulu-Dahunsi AT, Verma DK, Thakur M, Tripathy S (2021). Functional importance of bioactive compounds of foods with potential health benefits: a review on recent trends. Food Biosci.

[2] Samtiya M, Aluko RE, Dhewa T, Moreno-Rojas JM (2021). Potential health benefits of plant food-derived bioactive components: an overview. Foods..

[3] Wu MS, Aquino LBB, Barbaza MYU, Hsieh CL, De Castro-Cruz KA, Yang LL (2019). Anti-inflammatory and anticancer properties of bioactive compounds from Sesamum indicum L. A Rev Mol.

[4] Khochapong W, Ketnawa S, Ogawa Y, Punbusayakul N (2021). Effect of in vitro digestion on bioactive compounds, antioxidant and antimicrobial activities of coffee (Coffea arabica L.) pulp aqueous extract. Food Chem.

[5] Ardabilchi Marand M, Amjadi S, Ardabilchi Marand M, Roufegarinejad L, Jafari SM (2020). Fortification of yogurt with flaxseed powder and evaluation of its fatty acid profile, physicochemical, antioxidant, and sensory properties. Powder Technol.

[6] Manthey FA, Lee RE, Hall CA (2002). Processing and cooking effects on lipid content and stability of α-Linolenic acid in spaghetti containing ground flaxseed. J Agric Food Chem.

[7] Durante M, Lenucci MS, Gazza L, Taddei F, Nocente F, De Benedetto GE (2019). Bioactive composition and sensory evaluation of innovative spaghetti supplemented with free or α-cyclodextrin chlatrated pumpkin oil extracted by supercritical CO2. Food Chem.

[8] Hwang CF, Chen YA, Luo C, Chiang WD (2016). Antioxidant and antibacterial activities of peptide fractions from flaxseed protein hydrolysed by protease from Bacillus altitudinis HK02. Int J Food Sci Technol.

[9] Hadi A, Askarpour M, Salamat S, Ghaedi E, Symonds ME, Miraghajani M (2020). Effect of flaxseed supplementation on lipid profile: An updated systematic review and dose-response meta-analysis of sixty-two randomized controlled trials. Pharmacol Res.

[10] Wu Y, Wang H, Wang Y, Brennan CS, Anne Brennan M, Qiu C (2021). Comparison of lignans and phenolic acids in different varieties of germinated flaxseed (Linum usitatissimum L.). Int J Food Sci Technol.

[11] Prasad K, Khan AS, Shoker M (2020). Flaxseed and Its components in treatment of hyperlipidemia and cardiovascular disease. Int J Angiol.

[12] Edel AL, Aliani M, Pierce GN (2015). Stability of bioactives in flaxseed and flaxseed-fortified foods. Food Res Int.

[13] De Aguiar AC, Boroski M, Monteiro ARG, De Souza NE, Visentainer JV (2011). Enrichment of whole wheat flaxseed bread with flaxseed oil. J Food Process Preserv.

[14] Marpalle P, Sonawane SK, Arya SS (2014). Effect of flaxseed flour addition on physicochemical and sensory properties of functional bread. LWT Food Sci Technol.

[15] Yaver E (2023). Dephytinized flaxseed flours by phytase enzyme and fermentation: functional ingredients to enhance the nutritional quality of noodles. J Sci Food Agric.

[16] Montedoro G, Servili M, Baldioli M, Miniati E. Simple and hydrolyzable phenolic compounds in virgin olive oil. 1. Their extraction, separation, and quantitative and semiquantitative evaluation by HPLC. ACS Publications. American Chemical Society. 2002;40:1571-6.

[17] Committee AA of CCAM. Approved Methods of the American Association of Cereal Chemists. AACC. 2000;670. Available online: http://methods.aaccnet.org/toc.aspx.

[18] Mekni M, Flamini G, Garrab M, Hmida RB, Cheraief I, Mastouri M (2013). Aroma volatile components, fatty acids and antibacterial activity of four Tunisian Punica granatum L. Flower cultivars. Industr Crops Prod.

[19] Braca A, De Tommasi N, Di Bari L, Pizza C, Politi M, Morelli I (2001). Antioxidant Principles from Bauhinia tarapotensis. J Nat Prod.

[20] Oyaizu M. Studies on products of browning reaction–antioxidative activities of products of browning reaction prepared from glucosamine. JpJ Nutr. 1986;44:307-15.

[21] Monteiro MLG, Mársico ET, Junior MSS, Magalhães AO, Canto ACVCS, Costa-Lima BRC (2016). Nutritional profile and chemical stability of pasta fortified with Tilapia (Oreochromis niloticus) Flour. Plos One.

[22] Rekha MN, Chauhan AS, Prabhasankar P, Ramteke RS, Rao GV (2013). Influence of vegetable purees on quality attributes of pastas made from bread wheat (T. Aestivum). CyTA - J Food..

[23] Yaqoob N, Munir H, Aslam F, Naseer R, Kamal S, Hussain S (2021). Antioxidant potential and phenolic contents of various flaxseed cultivars from different agro-industrial regions. Pol J Environ Stud.

[24] Dhirhi N, Shukla R, Patel NB, Sahu E, Gendley T, Mehta N (2016). « lignan » - Antioxidant of linseed. Plant Arch.

[25] Morris DH. Flax: A Health and Nutrition Primer. Flax Council of Canada; 2007;144

[26] Dzuvor CKO, Taylor JT, Acquah C, Pan S, Agyei D (2018). Bioprocessing of functional ingredients from Flaxseed. Molecules.

[27] Zhang S, Chen Y, McClements DJ, Hou T, Geng F, Chen P (2023). Composition, processing, and quality control of whole flaxseed products used to fortify foods. Comprehen Rev Food Sci Food Safety.

[28] Cheng C, Yu X, McClements DJ, Huang Q, Tang H, Yu K (2019). Effect of flaxseed polyphenols on physical stability and oxidative stability of flaxseed oil-in-water nanoemulsions. Food Chem.

[29] Zhu F, Li J (2019). Physicochemical and sensory properties of fresh noodles fortified with ground linseed (Linum usitatissimum). LWT.

[30] Hao M, Beta T (2012). Development of Chinese steamed bread enriched in bioactive compounds from barley hull and flaxseed hull extracts. Food Chem.

[31] Kezimana P, Dmitriev AA, Kudryavtseva AV, Romanova EV, Melnikova NV (2018). Secoisolariciresinol Diglucoside of flaxseed and its metabolites: biosynthesis and potential for nutraceuticals. Front Genet.

[32] Waszkowiak K, Siger A, Rudzińska M, Bamber W (2020). Effect of roasting on flaxseed oil quality and stability. J Am Oil Chem’ Soc.

[33] Kaur P, Sharma P, Kumar V, Panghal A, Kaur J, Gat Y (2019). Effect of addition of flaxseed flour on phytochemical, physicochemical, nutritional, and textural properties of cookies. J Saudi Soc Agric Sci.

[34] Lee RE, Manthey FA, Iii CAH (2004). Content and stability of Hexane extractable lipid at various steps of producing macaroni containing ground flaxseed. J Food Process Preserv.

[35] Hrušková M, Svec I. Flax - Evaluation of composite flour and using in cereal products. Potravinarstvo. 2016;10:287-94.

[36] Marie H, Svec I (2017). Rheological characteristics of composite flour with linseed fibre – relationship to bread quality. Czech J Food Sci.

[37] Lipilina E, Ganji V (2009). Incorporation of ground flaxseed into bakery products and its effect on sensory and nutritional characteristics – a pilot study. J Foodserv.

